# The Seasonal Incidence of Slipped Capital Femoral Epiphysis: A Systematic Review and Meta-Analysis

**DOI:** 10.3390/children12060729

**Published:** 2025-05-31

**Authors:** Carter J. K. White, Jacob D. Kodra, Harshavardhan Bollepalli, Kai Yang, Scott E. Van Valin, Xue-Cheng Liu

**Affiliations:** 1 School of Medicine, Medical College of Wisconsin, Milwaukee, WI 53226, USA; cawhite@mcw.edu (C.J.K.W.);; 2 Division of Biostatistics, Institute for Health and Equity, Medical College of Wisconsin, Milwaukee, WI 53226, USA; 3 Department of Orthopedic Surgery, Children’s Wisconsin, Medical College of Wisconsin, Greenfield, WI 53227, USA

**Keywords:** SCFE, seasonal variation, pediatric orthopedics, incidence, cosinor regression, growth plate, physeal injuries, epidemiology

## Abstract

Background/Objectives: Slipped capital femoral epiphysis (SCFE) is a common adolescent hip disorder characterized by the posterior and inferior slippage of the femoral head through the growth plate. This study aimed to assess seasonal trends in the incidence of SCFE above 40° N latitude using cosinor regression analysis. Methods: A systematic review was conducted following Preferred Reporting Items for Systematic Reviews and Meta-Analyses (PRISMA) guidelines and registration in Prospero. A keyword search of PubMed, Scopus, Web of Science, and Ovid was conducted using terms related to SCFE and seasonal or monthly variation. Studies were included if they reported month-by-month SCFE incidence in pediatric populations. Studies were excluded if they had duplicated population cohorts. Data from studies conducted above 40° N were extracted for cosinor regression analysis. Quality assessment was performed using the JBI Critical Appraisal Checklist for Studies Reporting Prevalence Data. Results: A total of seventeen studies were included, amassing a total of 21,428 children analyzed. Six investigations reported monthly incidence data for populations > 40° N. The highest pooled monthly incidence was observed in August (482 cases). Alternatively, February was the month with the lowest SCFE incidence (293). Cosinor regression analysis demonstrated that SCFE occurs more frequently during early summer and fall months (*p* < 0.0001). Conclusions: SCFE incidence follows a significant seasonal trend in higher-latitude (>40° N) populations, with a consistent late-summer peak. Recognizing seasonal variation patterns may inform early diagnosis and intervention in SCFE, improving outcomes in pediatric physeal injuries.

## 1. Introduction

Slipped capital femoral epiphysis (SCFE) is the most common hip disorder in adolescents, marked by the displacement of the proximal femoral epiphysis posteriorly and inferiorly relative to the femoral neck [1,2,3,4,5]. The underlying causes of SCFE appear to be multifactorial, likely involving mechanical stress, hormonal factors, obesity, and rapid growth during puberty [6,7,8,9,10]. These injuries often present gradually with vague symptoms such as hip, groin, or knee pain, with any misdiagnosis having the potential for serious complications such as avascular necrosis, chondrolysis, leg length discrepancies, and early-onset osteoarthritis [11,12,13].

The incidence of SCFE varies globally, with previous reports suggesting approximately 4–10 cases per 100,000 children [14,15]. However, other works have also identified substantial geographical variability in SCFE occurrence, with higher incidences reported in North America and Western Europe compared to Asian and African countries [16,17,18]. Particularly, rates in Japan and Korea are very low. For example, Song et al. estimated the average annual incidence of SCFE to be at least 0.499 for males and 0.142 for females per 100,000 children aged 10 to 14 years old in South Korea—a reported incidence nearly half of that described amongst North American regions [19,20]. It has been suggested that this variation may be attributable to differences in genetic predisposition, environmental factors, and cultural practices across these regions. Separately, an emerging phenomenon in SCFE research underscores an apparent seasonal incidence pattern in which the rates of SCFE change throughout the year [19,20,21,22]. It is thought that this could be due to changes in sunlight exposure, hormones, activity levels, and velocity of growth [23,24]. Further, the literature suggests that this seasonal pattern also demonstrates geographical variation, potentially favoring environmental factors more than cultural influence [21]. Thus, both the geography and the season play a role separately.

The purpose of the present study was to assess seasonal trends in the incidence of SCFE above 40° N latitude using cosinor regression analysis. We hypothesized that SCFE would demonstrate a clear seasonal pattern and occur with the greatest incidence during the summer months.

## 2. Materials and Methods

This review was conducted in accordance with the Preferred Reporting Items for Systematic Reviews and Meta-Analyses (PRISMA, School of Public Health and Preventive Medicine, Monash University, Melbourne, Australia) guidelines. The study was registered in Prospero (CRD420251025725) to ensure transparency.

### 2.1. Search Strategy

A comprehensive keyword search was performed in March 2025 across the Web of Science, Scopus, PubMed, and Ovid databases without any year restriction. The specific search terms used in each database are outlined to ensure transparency and reproducibility in Supplementary Table S1.

### 2.2. Screening and Selection

The included studies were imported into the online software Rayyan (Qatar Computing Research Institute, Hamad Bin Khalifa University, Doha, Qatar). Duplicate studies were removed, and studies were initially screened for criteria fit by title and abstract. The studies that met the criteria were further screened by three separate reviewers, and conflicts were discussed and resolved by majority vote. Full-text screening was then performed, with disagreements again resolved by majority vote. While formal inter-reliability statistics (kappa coefficient) were not calculated, all final decisions were made by unanimous agreement following reconciliation discussions.

### 2.3. Data Extraction

Microsoft Excel (Microsoft Office, 2011; Microsoft, Redmond, WA, USA) was used to import data manually from included studies. The data extracted included authors, year, city/country of study population, degrees of latitude of study population, number of patients, if seasonality was reported to be a significant finding, and incidence by month. Three reviewers completed data extraction, and any discrepancies were resolved by repeated extraction.

### 2.4. Quality Assessment

The quality assessment of studies was performed with the Joanna Briggs Institute (JBI) Checklist for Prevalence or Incidence Studies [25]. This tool is specifically designed to assess studies reporting population-level estimates such as incidence, making it appropriate for the present analysis. The checklist evaluates domains such as the appropriateness of the sampling, the reliability of case identification methods, and the adequacy of statistical analysis. The fulfillment of all checklist items was not required for inclusion, provided that the data were judged to be sufficiently robust and relevant for quantitative synthesis.

### 2.5. Statistical Analysis

Studies that reported the incidence of SCFE by month were included for analysis. Studies were categorized by degrees of latitude (>40° N and <40° N). Only one study reported month-by-month data in locations < 40° N; thus, the pooled analysis only included data from locations > 40° N to reduce regional confounders. Given the exclusion of <40° N in statistical analysis, seasonality in these geographies cannot be tested using a cosinor model. All statistical analysis was performed by a biostatistician with extensive experience in meta-analyses. The included data were pooled utilizing cosinor regression analysis. This was performed in R 4.3.2 using a cosinor model [26,27]. Statistical significance was defined to be *p* < 0.05.

## 3. Results

### 3.1. Study Identification

**Figure 1:**
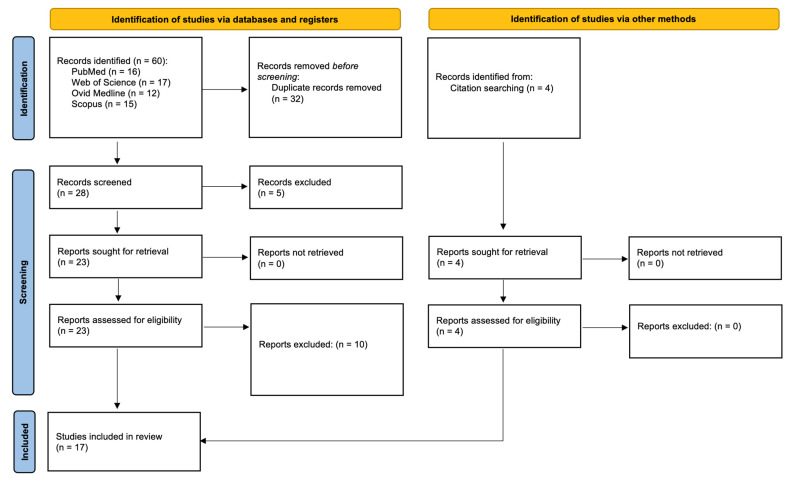
Flowchart according to PRISMA (Preferred Reporting Items for Systematic Reviews and Meta-Analyses) guidelines.

A systematic search of the Web of Science, Scopus, PubMed, and Ovid databases generated a total of 60 studies (Figure 1). After the removal of inter-database duplicates, 28 full-text articles remained. After title and abstract screening, 23 full-text articles remained. After full-text screening, 13 articles remained. Additionally, 4 studies were then added to these 13 from citation searching. This resulted in a total of 17 studies that were included for analysis.

### 3.2. Appraisal of Studies

All 17 studies included in this review were evaluated for methodological quality using the Joanna Briggs Institute Checklist for Prevalence and Incidence Studies [25]. All included studies demonstrated sufficient methodological rigor to justify inclusion. There were clear definitions of the study population, valid approaches to identifying SCFE cases, and appropriate handling of temporal data when reported. No studies were excluded based on quality concerns.

### 3.3. Data Pooling

The study characteristics of all eligible studies are summarized in Table 1. There were 12 studies > 40° N with statistically significant seasonal incidence data [22,28,29,30,31,32,33,34,35,36,37,38]. Alternatively, two studies > 40° N failed to identify significant differences in monthly SCFE variation [39,40]. Only one study was identified <40° N that achieved seasonal significance, but it did not report month-by-month incidence data [41]. There were two studies <40° N that did not report seasonal significance [42,43]. These studies also did not report month-by-month incidence data.

Of the 17 total studies included, 6 studies with populations >40° N included month-by-month incidence data that were viable for cosinor regression analysis. A summary of the monthly incidence of SCFE among studies analyzed using the cosinor model is displayed in Table 2. Considering that only one study below 40° N had month-by-month data, cosinor analysis was not performed for <40° N in order to maintain a homogenous cohort for analysis. Any study that did not report month-by-month data could not be included in the analysis.

**Figure 2:**
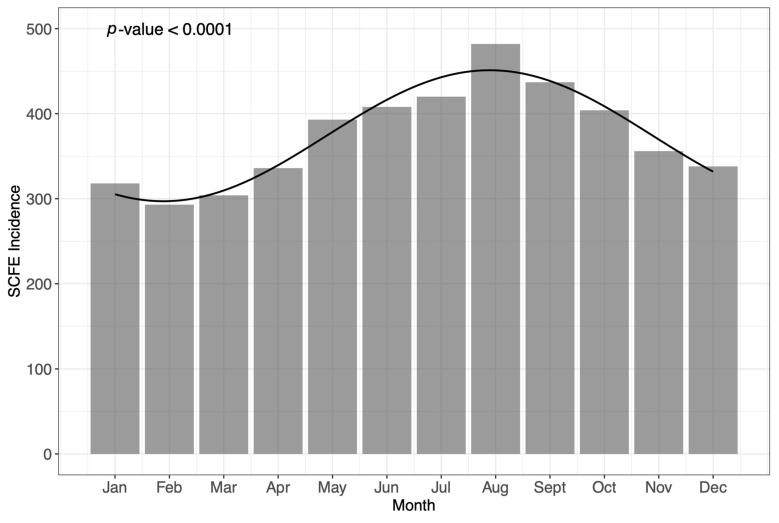
A graphical representation of pooled monthly incidence of SCFE across all eligible studies, with a linear regression line generated from cosinor analysis.

The pooled monthly incidence of SCFE across all eligible studies is seen in Figure 2 [28,32,33,34,35,37]. Each bar represents the incidence of SCFE by specific month, while the solid line is representative of the linear regression determined from cosinor analysis. The formula for the cosinor model is given as follows: Incidence = 374.08 − 77.02 × cos (2 π t/12 − 1.00), where t = 1 for January, t = 2 for February, and so on. Cosinor regression analysis indicated that seasonal variation in SCFE was statistically significant (*p* < 0.0001). This *p*-value represents the probability of observing the detected seasonal pattern if there were truly no underlying seasonal variations in SCFE incidence. A *p*-value this low strongly suggests that the observed seasonal fluctuation is unlikely due to random chance, providing robust evidence of a statistically significant cyclical trend in the timing of SCFE cases throughout the year.

## 4. Discussion

This meta-analysis confirms the presence of an SCFE seasonal incidence pattern, which peaks in the late-summer months at latitudes greater than 40° North latitude. Using cosinor regression modeling, we identified a statistically significant fluctuation in monthly cases, with a peak in August and a low point in February.

The current study builds upon a 2015 meta-analysis by Farrier et al., which reviewed available data on SCFE seasonality. Farrier similarly identified statistically significant variation in SCFE incidence by month [21]. This trend was most pronounced in populations living above 40° N latitude, where cases peaked in late summer; however, seasonal patterns in lower latitudes were less clear. These findings largely align with our updated meta-analysis, which uniquely focused on describing and quantifying SCFE seasonality using a cosinor statistical analysis model.

The present study was able to contribute greater statistical power with the addition of 3256 more patients from two new studies for analysis and provides a more refined estimate of the timing of seasonal variation. While the 2015 study showed some variability in peak months, our results reinforce the existence of an annual peak, most pronounced in August. The reasons behind this seasonal pattern are still not fully understood. Farrier et al. also proposed a potential connection between this seasonality and fluctuations in serum vitamin D levels, although this remains a topic of debate [10,46,47,48,49]. Previous case series studies, such as Elbeshry et al., asserted that there may be an association between vitamin D and the development of bilateral SCFE due to the role of vitamin D in bone growth [46]. Torres-Izquierdo et al., using a large database with over 35,000 matched patients, identified vitamin D deficiency as a significant risk factor for developing SCFE [50]. However, a recent study also probed this association further and showed that vitamin D did not have an association with sunlight levels in children [51]. Thus, the theory that sunlight exposure leads to vitamin D synthesis may not be the complete picture. Other hypothesized contributors include seasonal shifts in growth rates, physical activity, and body weight, which could contribute to increased mechanical stress on the growth plate. Mirwald et al. showed that height velocity in 8–18-year-olds peaks in the summer months, with 60% to 67% of annual height growth occurring during the summer months [52]. This rapid acceleration in height could place the growth plate under additional stress, potentially predisposing patients to SCFE.

Hosseinzadeh et al. and Lim et al. attempt to describe the biomechanical stress that increased weight places on the anatomic hip [53,54]. Further, BMI also has a long-known association with SCFE [23,55,56,57]. However, evidence suggesting that any one of these factors is directly causative of SCFE pathogenesis remains unproven.

The August peak in SCFE likely reflects a combination of these factors, while the February trough could be attributed to slower growth, less physical activity, or seasonal differences in how quickly families seek medical attention. A recent study from Fitzpatrick et al. identified that rates of missed primary care appointments increased by 0.72% for every 1° F decrease in daily maximum temperatures below 39° F [58]. This could be one reason that children in colder climates, such as those above 40° N, present less often in the winter, and may present later in summer with SCFE. Research into the biomechanical and pathophysiological aspects of SCFE remains limited, likely due to the complexity and difficulty of conducting such studies. Future studies could address the idea that the variation in incidence is likely multi-factorial. Many studies have focused on one aspect that may play a role; however, there has not been a study that has examined how much each factor plays into pathogenesis.

The majority of studies conducted below 40° N did not report detailed monthly data, limiting our ability to explore trends in lower-latitude populations. This highlights the need for more consistent and detailed temporal data in future SCFE research, especially from geographically diverse regions. Additionally, studies that focus on building an early monitoring system for SCFE development can use epidemiological data like that reported in this study to refine their predictions. Some studies have focused on the quantification of risk factors for SCFE, which can be useful in determining a patient’s risk profile [59]. With the rapid development of big-data models used to predict and prevent diseases, SCFE may be a condition that benefits from such models [60].

This study has reportable limitations. First, all of the included studies were conducted with a retrospective design, potentially contributing to biases in data reporting and extraction. Second, SCFE onset was inconsistently defined at times, allowing for some variability, which may influence our reported trends. Finally, although we aimed to include all studies reporting on SCFE seasonality, many did not provide usable month-by-month data, restricting our ability to increase the power of our analysis.

## 5. Conclusions

SCFE incidence follows a significant seasonal trend in higher-latitude (>40° N) populations, with a consistent late-summer peak. Recognizing seasonal variation patterns may inform early diagnosis and intervention in SCFE, improving outcomes in pediatric physeal pathology.

## Figures and Tables

**Table 1:** Study characteristics.

Study	Year	Number of Patients	Location	Significance in Seasonality?	Reported Monthly Data?
Ferguson et al. [30]	1931	55	New York 41° N	Yes	No
Andren et al. [31]	1958	67	Malmo, Sweden 44° N	Yes	No
Sorensen [32]	1968	61	Aarhus, Denmark 56° N	Yes	Yes
Hagglund/Hansson [33,44]	1984/1987	532	Southern Sweden 56° N	Yes	Yes
Loder et al. [34]	1990	177	Detroit, Michigan 44° N	Yes	Yes
Loder [35]	1996	1630	Worldwide	Yes > 40° N	Yes
Jerre et al. [39]	1996	175	Gothenburg, Sweden 57° N	No	No
Maffulli et al. [36]	2002	1103	Aberdeen, Scottland 57° N	Yes	No
Noguchi et al. [41]	2002	202	Japan 35° N (24–46°)	Yes	No
Brown [45]	2004	4690	Multiple States	Yes > 40° N, No < 40° N	No
Lehman et al. [38]	2006	1512	Multiple States	Yes	No
Benson et al. [42]	2008	183	New Mexico 36° N	No	No
Herngren et al. [37]	2017	379	Sweden (>40° N)	Yes	Yes
Loder et al. [28]	2019	10,350	United States	Yes > 40° N	Yes
Firth et al. [22]	2020	137 UK (70 SA)	Johannesburg, South Africa (26.2° S latitude) and London, UK (51.5° N latitude)	Yes	No
Ripatti et al. [40]	2023	159	Finland (>40° N)	No	No
Taşci et al. [43]	2024	720	~39° N (Türkiye, nationwide data)	No	No

**Table 2:** Monthly SCFE incidence among studies analyzing a latitude >40°N.

Study	Sorensen [32]	Hagglund/Hansson [33,44]	Loder et al. [34]	Loder [35]	Herngren et al. [37]	Loder et al. [28]	Total SCFE Incidence
Year	1968	1984/1987	1990	1996	2017	2019	
January	4	19	14	29	21	231	318
February	3	42	12	28	15	193	293
March	1	31	6	27	20	219	304
April	5	30	24	43	29	205	336
May	3	42	20	53	27	248	393
June	4	44	30	62	44	224	408
July	8	30	20	54	37	271	420
August	6	52	30	58	50	286	482
September	8	33	15	63	29	289	437
October	4	37	11	38	34	280	404
November	7	30	14	46	34	225	356
December	8	43	12	32	29	214	338

## Data Availability

These data were derived from the resources described and are available in the indicated databases.

## Supplementary Materials

The following are available online at https://www.mdpi.com/article/10.3390/children12060729/s1, Table S1: A comprehensive list of databases used and their respective keyword search queries.

## Abbreviations

The following abbreviations are used in this manuscript:

SCFE	Slipped Capital Femoral Epiphysis
PRISMA	Preferred Reporting Items for Systematic Reviews and Meta-Analyses
