# Supplementary material for: The Seasonal Incidence of Slipped Capital Femoral Epiphysis: A Systematic Review and Meta-Analysis
Source: Children (Basel). 2025 May 31;12(6):729. doi: 10.3390/children12060729 (PMC12190929; doi:10.3390/children12060729)
Supplement: Supplementary file 1 [file children-12-00729-s001.zip › children-3645725-supplementary.pdf]

Supplementary Materials

Table 1. A comprehensive list of databases used and their respective keyword search queries.

Database	Search Query
Web of Science	(Seasonal OR "Seasonal Variation" OR "Yearly Variation" OR "Temporal Variation" OR "Monthly Variation" OR "Annual Variation") AND (SCFE OR "Slipped Capital Femoral Epiphysis" OR SUFE OR "Slipped Upper Femoral Epiphysis")<br>Filters: English language
Scopus	TITLE-ABS-KEY ((seasonal OR "Seasonal Variation" OR "Yearly Variation" OR "Temporal Variation" OR "Monthly Variation" OR "Annual Variation") AND (scfe OR "Slipped Capital Femoral Epiphysis" OR sufe OR "Slipped Upper Femoral Epiphysis"))
PubMed	(seasonal OR "Seasonal Variation" OR "Yearly Variation" OR "Temporal Variation" OR "Monthly Variation" OR "Annual Variation") AND (scfe OR "Slipped Capital Femoral Epiphysis" OR sufe OR "Slipped Upper Femoral Epiphysis")
Ovid	((seasonal or "Seasonal Variation" or "Yearly Variation" or "Temporal Variation" or "Monthly Variation" or "Annual Variation") and (scfe or "Slipped Capital Femoral Epiphysis" or sufe or "Slipped Upper Femoral Epiphysis")).mp.<br>Search fields (mp): title, book title, abstract, original title, name of substance word, subject heading word, floating sub-heading word, keyword heading word, organism supplementary concept word, protocol supplementary concept word, rare disease supplementary concept word, unique identifier, synonyms, population supplementary concept word, anatomy supplementary concept word

JBI Checklist for Incidence/Prevalence Studies:

Study	1. Sample frame appropriate?	2. Sampling method appropriate?	3. Sample size adequate?	4. Subjects & setting detailed?	5. Data analysis sufficient coverage?	6. Valid methods for condition ID?	7. Condition measured reliably?	8. Statistical analysis appropriate?	9. Adequate response rate/handled?
Ferguson et al (1931)	Yes	Yes	Yes	Unclear	No	Yes	Yes	Yes	Unclear
Andren et al (1958)	Yes	Yes	Yes	Unclear	No	Yes	Yes	Yes	Unclear
Sorensen (1968)	Yes	Yes	Yes	Yes	Yes	Yes	Yes	Yes	Yes
Hagglund/Hansson (1984/1987)	Yes	Yes	Yes	Yes	Yes	Yes	Yes	Yes	Yes
Loder et al (1990)	Yes	Yes	Yes	Yes	Yes	Yes	Yes	Yes	Yes
Loder (1996)	Yes	Yes	Yes	Unclear	Yes	Yes	Yes	Yes	Yes
Jerre et al (1996)	Yes	Yes	Yes	Unclear	No	Yes	Yes	Yes	Unclear
Maffulli et al (2002)	Yes	Yes	Yes	Unclear	No	Yes	Yes	Yes	Unclear
Noguchi et al (2002)	Yes	Yes	Yes	Unclear	No	Yes	Yes	Yes	Unclear
Brown (2004)	Yes	Yes	Yes	Unclear	No	Yes	Yes	Yes	Unclear
Lehman et al (2006)	Yes	Yes	Yes	Unclear	No	Yes	Yes	Yes	Unclear
Benson et al (2008)	Yes	Yes	Yes	Unclear	No	Yes	Yes	Yes	Unclear
Herngren et al (2017)	Yes	Yes	Yes	Yes	Yes	Yes	Yes	Yes	Yes
Loder et al (2019)	Yes	Yes	Yes	Yes	Yes	Yes	Yes	Yes	Yes
Firth et al (2020)	Yes	Yes	Yes	Unclear	No	Yes	Yes	Yes	Unclear
Ripatti et al (2023)	Yes	Yes	Yes	Unclear	No	Yes	Yes	Yes	Unclear
Taşci et al (2024)	Yes	Yes	Yes	Unclear	No	Yes	Yes	Yes	Unclear
